# Disposal of iNKT cell deficiency and an increase in expression of SLAM signaling factors characterizes sarcoidosis remission: a 4-year longitudinal study

**DOI:** 10.1186/s12931-014-0091-4

**Published:** 2014-08-21

**Authors:** Katarina Osolnik, Matija Rijavec, Peter Korosec

**Affiliations:** University Clinic of Respiratory and Allergic Diseases Golnik; Laboratory for Clinical Immunology & Molecular Genetics, Golnik 36, Golnik, 4204 Slovenia

## Abstract

**Background:**

Invariant NKT (iNKT) cells are regulatory lymphocytes that may be important in disorders with increased Th1 responses. We utilized a 4-year longitudinal observational study of iNKT cells and SLAM signaling pathway factors, which are important for iNKT development in patients with newly diagnosed sarcoidosis.

**Methods:**

Detailed clinical, functional, and radiographic evaluation and determination of iNKT peripheral blood cell counts and expression of SLAM signaling factors was carried out at presentation and after 3 months, 1 year, and 4 years of disease follow-up in 29 patients with pulmonary sarcoidosis. At presentation, we also evaluated the frequencies of pulmonary BALF iNKT cells. We also included 37 control subjects.

**Results:**

We demonstrated a marked deficiency of blood and lung iNKT cells and decreased expression of SLAM signaling factors in patients with newly diagnosed sarcoidosis. During 4 years of disease follow-up, there was a significant increase in blood iNKT cell numbers and in expression of SLAM signaling factors, mainly *SLAMF1*, *SLAMF6*, and *FYN*. This increase clearly correlated with improvement in patients’ clinical symptoms. At the 4-year endpoint, the disease had gone into remission in the great majority of patients and thus also iNKT cell deficiency. Moreover, at the 4-year endpoint iNKT level reached the iNKT level of the control subjects.

**Conclusions:**

Our longitudinal study showed that a disposal of iNKT deficiency in parallel with an increase in expression of SLAM signaling factors characterizes the clinical remission of sarcoidosis.

## Introduction

In contrast to both conventional T lymphocytes and other types of Tregs, invariant natural killer T (iNKT) cells comprise a unique subgroup of immunoregulatory T lymphocytes that are restricted by the non-classical MHC class I molecule CD1d and expression of the invariant Vα24–Jα18 T cell receptor (TCR) preferably paired with Vβ11 [1-6]. Furthermore, iNKT cells can either upregulate or downregulate cell-mediated immunity and thus these cells could have diverse influences in various disease models [5-8].

Sarcoidosis is the most common interstitial lung disease in the western world. It frequently involves organs other than the lungs. This multisystem disorder features the presence of CD4^+^ T cells that differentiate into Th1-like cells and amplify the local immune response [9-11]. Despite much research over the past decade, the cause of the Th1-biased hyperactive response remains unknown. Ho et al. [12] first hypothesized that the loss of immunoregulation by iNKT cells could explain amplified and persistent T cell activity. This hypothesis was based on NOD mice that are deficient for iNKT cells. These mice are generally healthy, but they are predisposed to disorders with increased Th1 responses [13]. Ho reported a significant decrease in iNKT cell frequency in the peripheral blood and BALF of sarcoidosis patients. We recently confirmed the deficiency of iNKT cells in BALF of corticosteroid-naive sarcoidosis patients [14]. Nevertheless, these association studies examined only one sample from each patient and, despite the fact that the iNKT cell defect was evident at some disease stages, there are currently no data about the nature of this defect or whether iNKT cells could be important for the long-term course of the disease. A more definitive study would have to closely follow the progress of iNKT cells over a longer period. Another question that arises is whether the iNKT cell defect is also affected by changes in iNKT cell development, which is highly dependent on signaling via the SLAM family of surface receptors [5,15-17]. For this reason, we followed up blood iNKT cells and expression of SLAM signaling factors together with detailed clinical data in 29 patients with newly diagnosed pulmonary sarcoidosis over 4 prospective years.

## Methods

### Study subjects

**Figure 1:**
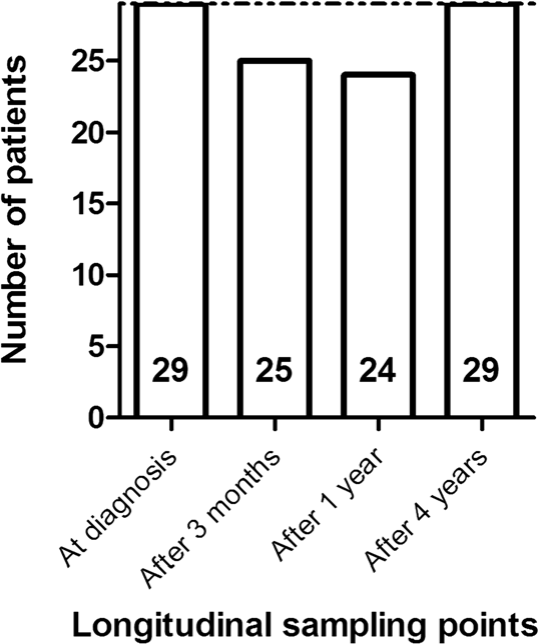
**The number of patients included with sarcoidosis at each longitudinal time point for cellular iNKT and SLAM expression analysis.**

Twenty-nine patients (mean age 38 years; range 25–74 years; 14 women, Caucasians) with newly diagnosed, histologically confirmed pulmonary sarcoidosis were recruited in a prospective manner from the Golnik University Clinic and first examined at diagnosis and then compared at 3 months, 1 year, and 4 years (mean after 4.2 years; range 3.5–4.6 years). Five subjects had Löfgren syndrome (Table 1). For iNKT and SLAM analysis, four patients were missing at 3 months and five at 1 year (Figure 1). We also included 28 healthy control subjects (mean age 39 years; range 20–62 years; 15 women); 15 control subjects were paired-sampled in a 3-month interval. The analysis involving BAL included an additional nine control subjects without any pulmonary morbidity (mean age 47 years; range 27–84 years; five women) and normal lung function tests. Some of these bronchoalveolar lavage (BAL) controls have already been published [14]. All subjects gave their written consent and the study was approved by the Slovenian National Medical Ethics Committee.

**Table 1:** **Results of chest radiographic stages, serum angiotensin–converting enzyme (sACE), lung function testing, and organ involvement in patients with newly diagnosed pulmonary sarcoidosis**

	**Sarcoidosis**
	**(** ***N*** **= 29)**
Chest radiographic stage*	
Stage I, *n* (%)	8 (28)
Stage II, *n* (%)	19 (66)
Stage III, *n* (%)	2 (7)
**Lung function**	
% predicted FEV1	92 (43–114)
% FVC	94 (51–118)
% DLCO	92 (46–122)
sACE (μkat/L) **	1.0 (0.1–4.4)
**Organ involved** ^**†**^	
Lung involvement, *n* (%)	29 (100)
Multisystem, *n* (%)	16 (55)
Erythema nodosum, *n* (%)	5 (17)
(Löfgren syndrome, *n* (%))	5 (17)^†^
Skin, *n* (%)	6 (21)
Liver, *n* (%)	3 (10)
Lymphatic glands, *n* (%)	2 (6)
Parotid glands and spleen, *n* (%)	1 (3)

Lung function and sACE data are presented as medians with the range.

*Standard radiographic staging consists of five stages: Stage 0 = normal, Stage I = bilateral hilar lymphadenopy, Stage II = BHL and parenchymal infiltration, Stage III = radioparenchymal infiltration without BHL, and Stage IV = irreversible fibrosis with loss of lung volume.

**sACE was determined by the kinetic method. Our normal values for sACE are 0.33–1.17 μkat/l. Sixteen (52%) patients had higher-than-normal concentrations of sACE.

^†^All patients with erythema nodosum included those fulfilling the criteria of Löfgren syndrome (plus bilateral hilar adenopathy and arthralgia).

### Chest radiographs, functional and clinical data

Pulmonary disease was evaluated at presentation and at the follow-up by chest radiography and pulmonary function testing in order to determine the disease course. FEV1, FVC, and DLCO were used to assess the presence of lung function impairment. All patients were also assessed for extrapulmonary disease and serum angiotensin-converting enzyme (sACE; Table 1).

### Bronchoalveolar lavage

BAL differential cell count and BAL TBNK immunophenotyping were performed at diagnosis, as previously described [14,18]. In brief: we instilled seven aliquots of 20 ml and one of 10 ml, up to a total volume of 150 ml of saline. After each instillation, the aliquot was immediately recovered, pooled, and thereafter processed for cytological and flow cytometry analysis. Cytospin preparations were stained according to the May–Grünwald–Giemsa and Papanicolaou methods and differential cell count was performed by counting 200 cells.

For TBNK immunophenotyping, the BALF was strained through a 70 mm cell strainer (BD Biosciences), centrifuged and resuspended in Haemaccel (Behringwerke, Marburg, Germany), and incubated with the respective mAbs (CD3, CD4, CD8, CD14, CD19, CD45, CD16/56, BD Biosciences, San Jose, CA, USA) for 15 min, followed by lysing, washing, and fixation. Labeled cells were analyzed by FACSCalibur and SimulSET software (BD Biosciences).

### iNKT cells

Invariant NKT (CD3 + Vα24-Jα18 + Vβ11+) were identified by using mAb against CD3, Vα24-Jα18 (clone 6B11) T cell receptor (TCR; all from BD Biosciences), and Vβ11 TCR (Immunotech, Marseille, France) [14,18]. Absolute iNKT counts were performed in combination with routine TruCOUNT tubes and TriTEST CD3/CD4/CD45 staining (all from BD Biosciences).

The frequencies of BALF iNKT cells were established at diagnosis and the cells were prepared alongside cells for TBNK immunophenotyping. First, the BALF cells were incubated with the respective iNKT mAbs for 15 min, followed by lysing, washing, and fixation, and then analyzed using FACSCalibur and CellQuest software (BD Biosciences). The BALF iNKT cells in patients were expressed as % of BALF T lymphocytes and were also compared with frequencies of BALF iNKT cells in control subjects.

Invariant NKT cells in peripheral blood were first examined at diagnosis and then compared at 3 months, 1 year, and 4 years for each patient. They were also compared with control subjects. Whole blood with EDTA anticoagulant was first incubated with iNKT mAbs for 15 min, followed by lysing, washing, and fixation, and analyzed using FACSCalibur and CellQuest software (BD Biosciences). The frequencies of blood iNKT cells were expressed as % of blood T lymphocytes. The parallel blood sample for absolute count was incubated with standardized TriTEST CD3/CD4/CD45 mAbs in TruCOUNT tubes followed by lysing and analyzing of T lymphocyte absolute numbers by FACSCalibur and MultiSET software (BD Biosciences). The absolute number of iNKT cells in whole blood was calculated by multiplying the frequencies of blood iNKT cells expressed as the proportion of blood T lymphocytes with the absolute number of blood T lymphocytes, and this was expressed as the number of iNKT cells per μl of whole blood.

### SLAM signaling factors gene expression

Whole blood was collected in PAXgene Blood RNA Tubes (PreAnalytiX GmbH, Hombrechtikon, Switzerland) at diagnosis and then at 3 months, 1 year, and 4 years for each patient and also in control subjects and stored at −30°C until further processing. Total RNA was isolated from blood samples using the PAXgene Blood RNA Kit (PreAnalytiX GmbH) and quantified by a Qubit fluorometer using the Quant-iT™ RNA Assay Kit (Invitrogen Corporation, Carlsbad, CA, USA) according to the manufacturer’s instructions. A reverse transcription reaction was performed using a High Capacity cDNA Reverse Transcription Kit (Applied Biosystems, Foster City, CA, USA).

Real-time PCR was performed on an ABI PRISM 7500 Real-Time PCR System (Sequence Detection System instrument equipped with SDS version 1.3.0 software; Applied Biosystems).

The human GAPD (GAPDH) Endogenous Control (Applied Biosystems) with the MGB probe labeled with FAM at the 5′ end was used for normalization of the target genes. Primers and probes for the genes of interest (*SLAMF1*, *SLAMF6*, *FYN*, and SLAM adaptor protein (SAP) (*SH2D1A*)) were also supplied by Applied Biosystems as TaqMan® Gene Expression Assays with the MGB probes labeled with FAM at the 5′ end (the assay IDs are Hs00158978_m1, Hs00941607_m1, Hs00234150_m1, and Hs00372941_m1, respectively). PCR reactions were set up in separate tubes with TaqMan Universal PCR Master Mix (Applied Biosystems) at default thermal conditions. All measurements were performed in triplicate for each time point and relative expression was analyzed using the ΔΔCt method.

### Statistical analysis

Data distribution was evaluated using the D’Agostino–Pearson test. Because the majority of data were not normally distributed, we performed a Wilcoxon or Mann–Whitney test. A chi-square test was used on binominal data. Data were expressed as median and range, if not otherwise specified. Probability values of *P* < 0.05 were accepted as significant. Analyses were performed using GraphPad Prism (GraphPad Software, La Jolla, CA, USA).

## Results

### Monitoring of pulmonary and extrapulmonary disease and treatment

**Figure 2:**
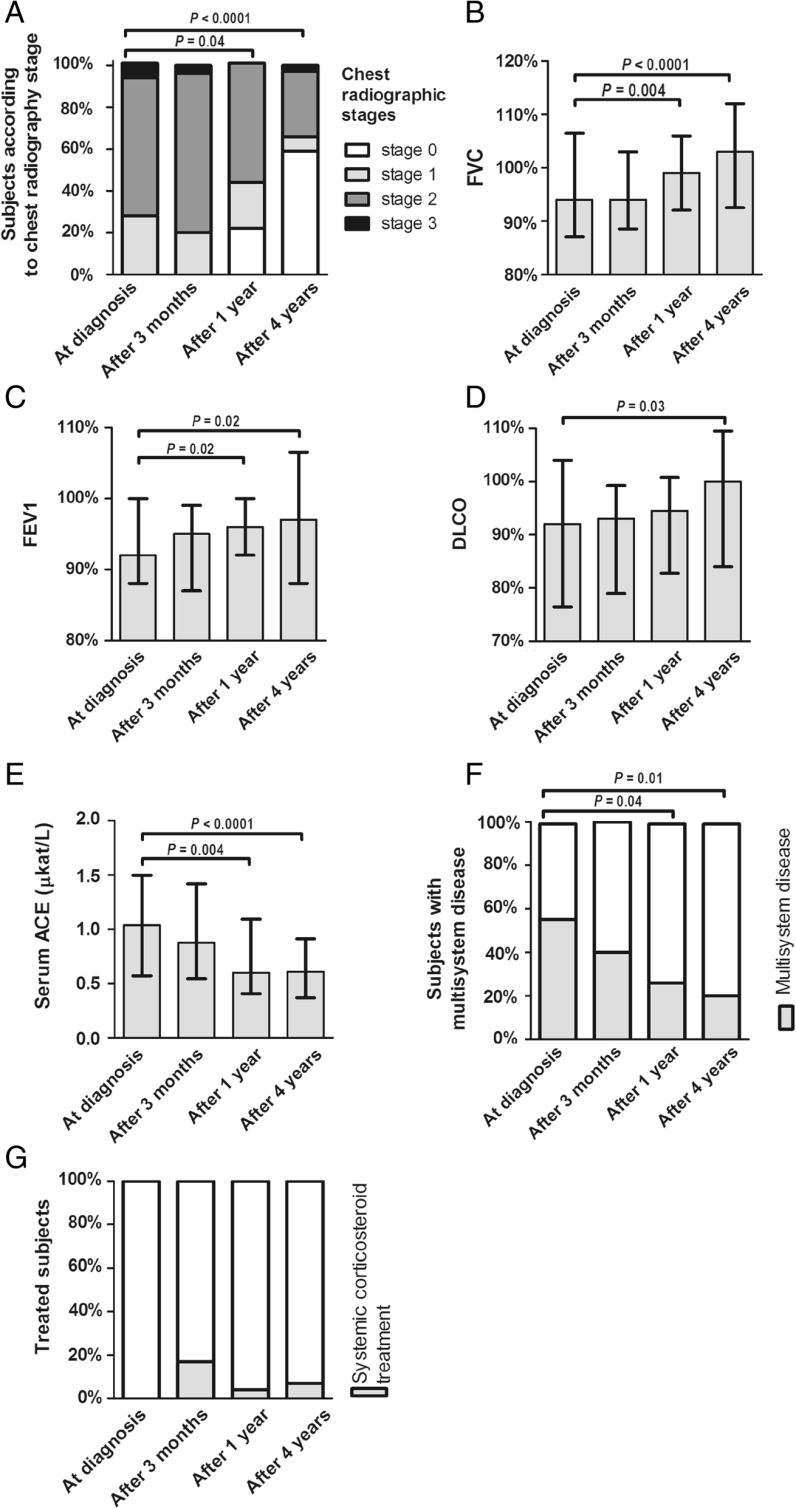
**Radiographic, functional, clinical, and treatment data in newly diagnosed patients with sarcoidosis and then over 4 years of disease follow-up: (A) chest radiographic stages, (B, C, D) lung functions, (E) serum ACE levels, (F) manifestations of granuloma in organs other than the lungs, and (G) systemic corticosteroid treatment.** The data measured are presented with the median and interquartile range.

At diagnosis, nine patients showed chest radiographic stage I, 19 showed stage II, and two showed stage III (Table 1). At 3 months, the stages were highly comparable. However, after 1 year there was a significant improvement in radiographic chest stages (six showed stage 0), and even more after 4 years, when 17 patients showed stage 0, two stage I, nine stage II, and one stage III (Figure 2A). The pulmonary function data also significantly improved, with a median increase of predicted FEV1 from 92 to 97%, FVC from 94 to 103%, and DLCO from 92 to 100% after 4 years of disease follow-up (Figure 2B–D). There was also a significant decrease of sACE (from 1 to 0.6 μkat/l; Figure 2E) after 4 years of disease follow-up.

At presentation, 16 patients showed extrapulmonary disease (Table 1). At follow-up there was a decrease to 12 patients at 3 months, to eight at 1 year, and finally after 4 years to six patients (four with skin involvement and two with liver involvement) (Figure 2F).

Among the 29 patients, only five needed initial treatment with oral corticosteroids. At 1 year only one patient remained on treatment; later, in the 3rd year, the disease again progressed in another patient, and so finally after 4 years two patients that seemed to have chronic disease needed corticosteroid treatment (Figure 2G).

### Monitoring of iNKT cells

**Figure 3:**
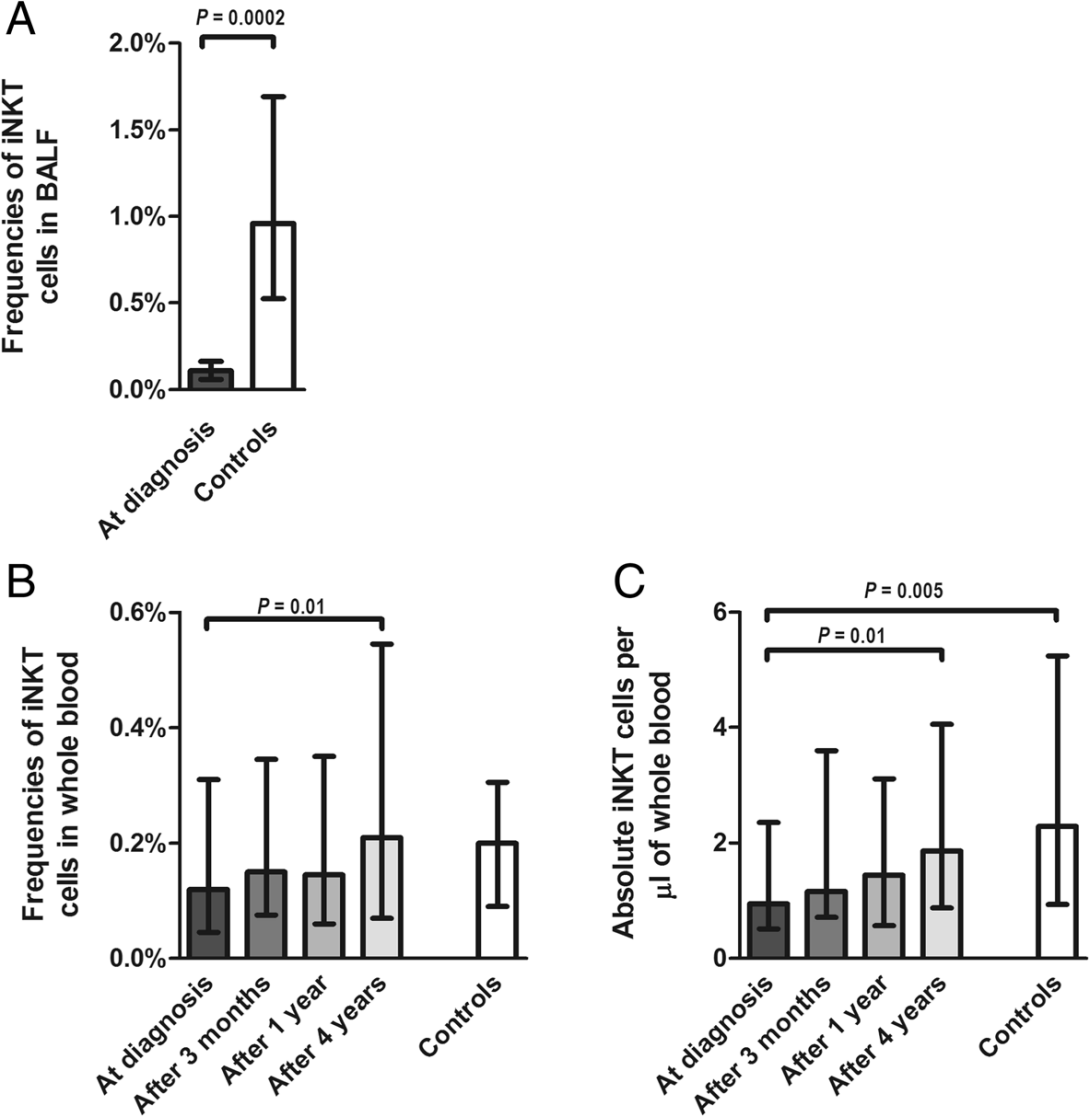
**Frequencies (A) of BALF iNKT (CD3** ^**+**^ **Vα24-Jα18-Vβ11) cells in newly diagnosed sarcoidosis patients and in comparison to BALF of the control subjects; Frequencies (B) and absolute counts of iNKT cells in whole blood (C) of the same patients over 4 years of disease follow-up and in comparison to the healthy control subjects.** Data are presented with the median and interquartile range.

The frequencies of BALF iNKT cells were determined only at the diagnosis and they were compared with the BALF of control subjects. The median (range) BALF iNKT cell frequency was 0.11% (0–2.55), whereas in controls the iNKT frequencies were about 10 times higher (0.96%; 0.2–2.51; Figure 3A). The accompanying analysis of BALF cell counts and TBNK immunophenotyping are presented in Table 2. BALF of sarcoidosis patients showed a higher lymphocyte total cell count and CD4/CD8 T ratio with higher CD4 and lower CD8 T lymphocyte subset frequencies, but comparable CD3 T and B lymphocyte, NK cell, and nonbiased NKT cell frequencies.

**Table 2:** **Bronchoalveolar lavage fluid (BALF) cell counts and T lymphocytes, B lymphocytes, NK, and NKT-like cells in BALF from patients with sarcoidosis sampled upon diagnosis and control subjects**

**BALF cell counts**	**Sarcoidosis** **(** ***N*** **= 29)**	**Controls** **(** ***N*** **= 9)**	***p*** **value**
Total cells (×10^5^ cells/ml)	1.2 (0.5–3.2)	1.00 (0.4–2.2)	n.s.
Neutrophils (%)	3 (1–6)	2 (1–22)	n.s.
Eosinophils (%)	1 (1–4)	1 (1–2)	n.s.
Macrophages (%)	68 (24–93)	87 (69–95)	0.003
Lymphocytes (%)	29 (6–76)	9 (3–14)	0.0005
**% of lymphocytes**			
T lymphocytes (CD3^+^)	89 (61–97)	86 (60–97)	n.s.
T helper (CD3^+^CD4^+^)	73 (37–90)	53 (27–80)	0.008
T cytotoxic (CD3^+^CD8^+^)	11 (4–39)	23 (13–57)	0.002
B lymphocytes (CD19^+^)	0 (0–3)	0 (0–2)	n.s.
NK cells (CD3^−^CD16/56^+^)	2 (1–32)	2 (0–4)	n.s.
nonbiased NKT cells (CD3^+^CD16/56^+^)	3.5 (1–11)	3 (2–5)	n.s.
CD4/CD8 ratio	7.29 (1–22.5)	2.2 (0.5–6.2)	0.002

Data are presented as medians with the range.

n.s. = non significant.

**Figure 4:**
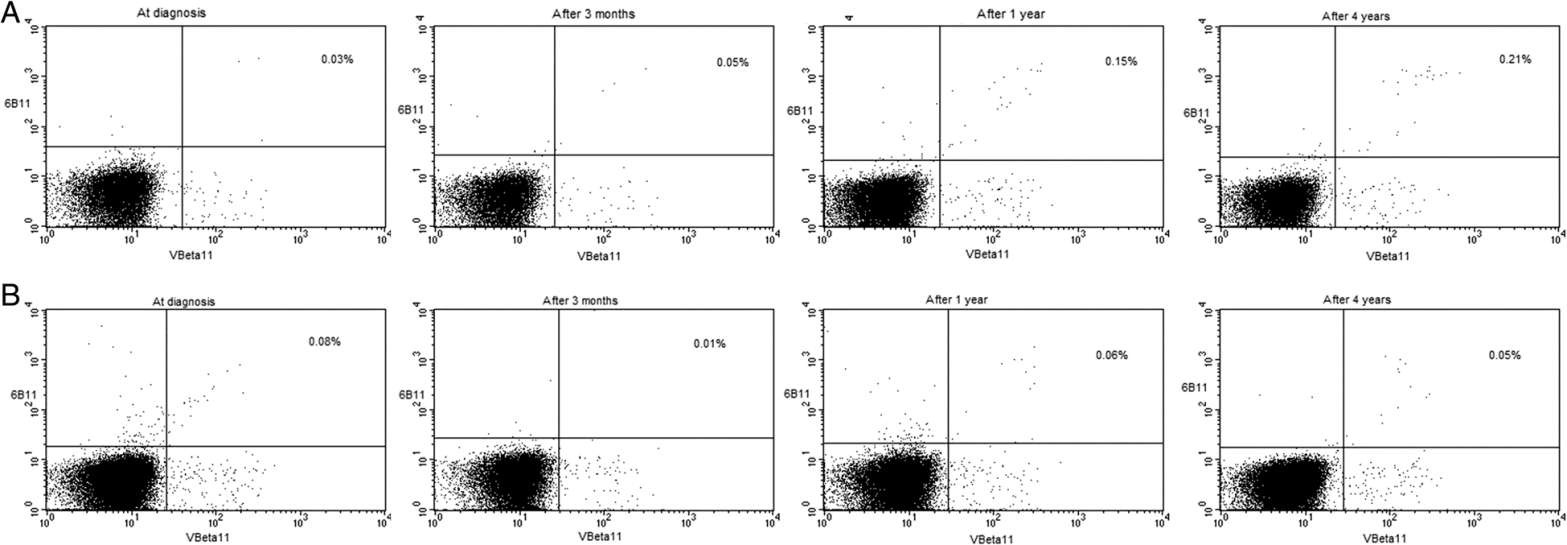
**Representative flow cytometry dot plots of iNKT cell analyzes over 4 years of disease follow-up (A) in a sarcoidosis patient with disease remission and (B) in a sarcoidosis patient with chronic disease.**

In peripheral blood, iNKT cells were closely monitored from the diagnosis of sarcoidosis over 4 prospective years. These data were also compared with iNKT cells in control subjects; in some of the controls iNKT cells were also monitored over 3 months. The median (range) frequency of blood iNKT cells at diagnosis was 0.12% (0.02–0.58), at 3 months 0.15% (0.01–0.76), at 1 year 0.15% (0.02–0.79), and after 4 years 0.21% (0.01–1.3) (Figure 3B). The median (range) absolute numbers of blood iNKT cells at diagnosis were 0.94 cells/μl (0.12–7.47), at 3 months 1.16 cells/μl (0.00–10.11), at 1 year 1.45 cells/μl (0.26–11.2), and after 4 years 1.87 cells/μl (0.14–15.7; Figure 3C). Thus after 4 years iNKT cell frequencies and absolute count significantly increased, and became comparable with control subjects (0.2%; 0.03–1.4 and 2.29 cells/μl; 0.41–12.3). Representative dot plots of iNKT cell analysis over 4 years of disease follow-up show a sarcoidosis patient with disease remission (Figure 4A) and a sarcoidosis patient with chronic disease (Figure 4B).

Monitoring of blood iNKT cells in 15 controls at the 3-month interval showed no differences, either in frequencies (0.25% (0.03–1.44) vs. 0.20% (0.03–1.42)) or in absolute counts (4.7 cells/μl (0.41–12.3) vs. 5 cells/μl (0.44–12.30)). Similarly, when we followed the frequencies and absolute count of blood T lymphocytes in sarcoidosis patients, we did not find any significant differences (at diagnosis vs. 4 years: 67% (39–86) vs. 70% (42–89) and 860 cells/μl (479–1832) vs. 1019 cells/μl (311–2416)).

### Monitoring the expression of SLAM signaling factors

**Figure 5:**
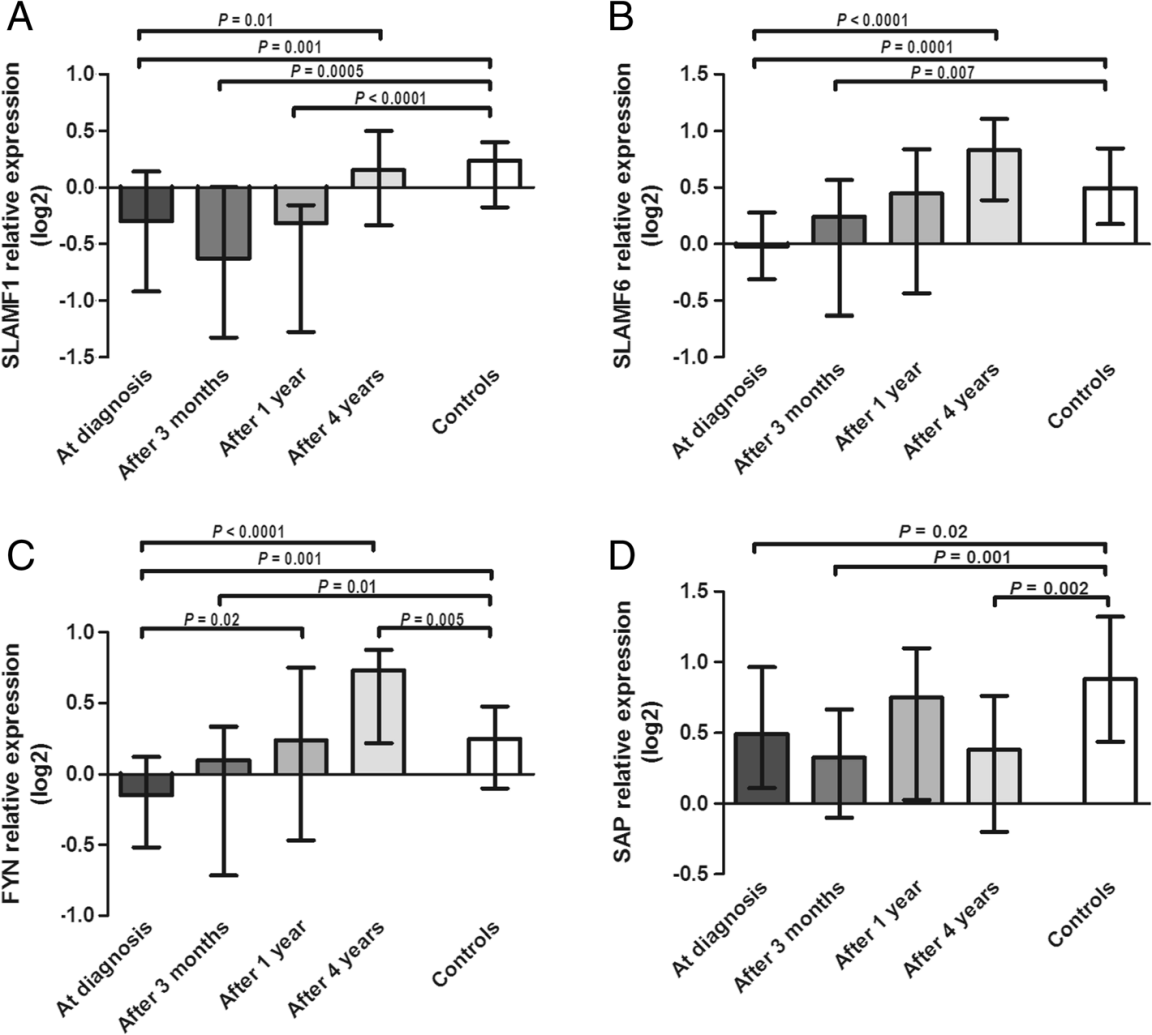
**Relative expression levels of genes (A)** ***SLAMF1*** **, (B)** ***SLAMF6*** **, (C)** ***FYN*** **, and (D)** ***SAP*** **in whole blood of patients with sarcoidosis over 4 years of disease follow-up and in comparison to healthy control subjects.** Data are presented with the median and interquartile range.

The expression of SLAM signaling factors *SLAMF1*, *SLAMF6*, *FYN,* and *SAP* was monitored in patients’ whole blood at the same time points as iNKT cells. *SLAMF1* expression remained similarly low after 3 months and 1 year of disease follow-up, but after 4 years it significantly increased and became comparable with the level in control subjects (Figure 5A). *SLAMF6* expression demonstrated a moderate increase through 3 months and significantly through 1 to 4 years of follow-up, and it was already comparable to controls after 1 year (Figure 5B). A very similar significant increase was also shown for *FYN* expression (Figure 5C). *SAP* expression varied through 3 months and 1 year of disease follow-up, and after 4 years it remained at the same level as at diagnosis and still lower than in control subjects (Figure 5D).

## Discussion

This study confirmed a marked blood and lung deficiency of invariant NKT cells in newly diagnosed patients with pulmonary sarcoidosis. It also showed that this deficiency is associated with decreased expression of the SLAM signaling factors that are important for development of these immunoregulatory T cells. Next, we closely followed the progress of blood iNKT cells and the expression of SLAM factors in sarcoidosis patients over a 4-year period. During longitudinal disease follow-up, there was a significant and gradual increase in iNKT cell numbers and expression of SLAM signaling factors. This increase correlated with improvement in patients’ overall radiographic, functional, and clinical symptoms, and finally at the 4-year endpoint the disease had gone into remission in the great majority of patients and thus also iNKT cell deficiency.

A causative association between iNKT cells and Th1-biased disorders such as sarcoidosis is based on the hypothesis that an iNKT cell deficiency affects their Th1-related immunoregulatory role. The most direct evidence for this hypothesis comes from animal models, such as NOD mice with lower iNKT cell numbers, which are characterized by a Th1-biased hyperactive response, and in which the adoptive transfer of iNKT cells has been shown to prevent the development of Th1-biased disorders [19-22]. Unfortunately, in the case of sarcoidosis, no reliable animal model is available; therefore only clinical analyses of iNKT cells can be studied. One of the key questions that arose with the first evidence of iNKT cell deficiency in sarcoidosis [12,14] was whether the peripheral blood deficiency reflects the systemic state or is simply a consequence of iNKT compartmentalization; that is, the lack of blood iNKT cells might be due to sequestration of these cells in the compartments of disease activity. It seems that compartmentalization is very unlikely because the deficiency of blood iNKT cells was clearly evident alongside the iNKT deficiency in airways [12, 14, and current report] as well as in the sarcoid mediastinal lymph nodes [23] and in granulomatous lesions of cutaneous sarcoidosis [24]. In contrast, only one report demonstrated increased *Vα24 TCR* expression by RT-PCR in the lymph nodes of sarcoidosis patients in comparison to the lymph nodes of lung-cancer patients [25]. Nevertheless, this observation was not confirmed by methodologically more stringent cellular approaches, which was the case for other reports [12,14,24].

In contrast to previous studies [12,14,24], this study benefited from long-term longitudinal sampling and detailed patient follow-up. Importantly, we closely followed the longitudinal progress of blood iNKT cells and showed that the increase in iNKT cells clearly correlated with patients’ clinical improvement. Moreover, after 4 years, when the disease resolved in the great majority of patients, blood iNKT levels reached the iNKT levels of the control subjects. Unfortunately, BAL sampling was carried out only at diagnosis and, because the disease was mostly resolved after 4 years, it did not seem ethical to perform another bronchoscopy and thus obtain a paired pulmonary sample. The observations that the restoration of iNKT cells could contribute to disease resolution also support the idea of possible therapeutic targeting of iNKT cells. Namely, clinical trials have shown that administering iNKT cell mitogens is well tolerated by patients and this can result in the expansion of residual iNKT cell populations [26]. In addition, this study did not show any differences concerning the nonbiased CD3 + CD16/56+ NKT cells in sarcoidosis patients, which is similar to previous reports [14,18].

It is not known what causes iNKT cell defects in sarcoidosis. In particular, iNKT cell development is highly dependent on SLAM signaling factors. SLAMF1 and its homologue SLAMF6 are type 1 plasma membranes and, in response to ligation, Src tyrosine kinase FYN is recruited to SAP [27]. In NOD mice a defect in SLAMF1 expression is responsible for the lower iNKT cell numbers [28]. Furthermore, mice lacking SAP completely lack iNKT cells, yet conventional T cells are present in normal numbers. In patients with X-linked lymphoproliferative disease (defect in the SH2D1A gene, which encodes the SAP molecule) development of iNKT cell is also absent [17]. However, a significant difference between human and mouse iNKT cells development is related to their place of maturation. In mice, mature iNKT cells develop in the thymus. In contrast, the equivalent development steps in humans are not completed in the thymus, but are completed in the periphery—or, at least, mature NKT cells are extremely rare in the human thymus, in contrast to their frequency in blood [29,30]. Due to the importance of the periphery for iNKT development in humans, it was reasonable to monitor the expression of SLAM signaling factors in the periphery of sarcoidosis patients. We showed that their expression upon disease presentation was much lower than in the control subjects, suggesting that impaired iNKT development might be the cause for the iNKT cell deficiency. Furthermore, during the course of the disease the expression of *SLAMF1*, *SLAMF6*, and *FYN* factors increased significantly, and this increase correlated with the increase in the iNKT cell numbers and the clinical remission of the disease.

The limitation of expression analysis was that SLAM family receptors are also expressed on T cells, B cells, and dendritic cells and SLAM adaptor protein (SAP) on T and NK cells [31]. However, only development of iNKT cells, but not other hematopoietic cells, is absent in patients with X-linked lymphoproliferative disease (SAP defect) [17] and SAP-deficient mice [16,17] or reduced in NOD [19-22], SLAMF1/SLAMF6 double mutant chimeras [27] and in Fyn deficient mice [32,33]. Because of that specific SLAM cellular development effect we believe that the measurement and quantitative follow-up of SLAM expression in whole blood sample is specific enough for their association with iNKT cells and also a possible reason for iNKT cell deficiency.

The heterogeneity among patients with sarcoidosis is well known [9]. However, the majority of patients with sarcoidosis have a remission within 5 years after diagnosis, with few or no consequences [9-11]. Furthermore, a recurrence after 1 or more years of remission is uncommon and affects less than 5% of patients, despite the fact that recurrent disease may develop at any age and in any organ [10,11]. Conversely, up to one-third of patients show unrelenting chronic disease that can lead to clinically significant organ impairment [10,11]. The incidence of chronic disease varies throughout the world, with the highest peak and severity in black Americans [10]. In our study, only two patients seem to have had chronic disease, and in all other patients the remission was clearly evident within 4 years. Furthermore, only five patients needed corticosteroid treatment up to the 1st year, and after 1 year only one chronic patient remained continually on treatment and another started again in the 3rd year. Löfgren syndrome—an acute presentation consisting of arthralgia, erythema nodosum, and bilateral hilar adenopathy—was exhibited in five patients. Obviously, our study group represents patients with pulmonary sarcoidosis that have a good prognosis. Therefore, the iNKT cellular and SLAM expression results and implication should be predisposing only for patients with such a reversible clinical phenotype. We also did not separately analyze chronic or Löfgren syndrome patients because those subgroups were too small.

## Conclusion

In conclusion, we showed that the deficiency of iNKT cells and decreased expression of SLAM signaling factors constitute a significant immunoregulatory T cell abnormality in sarcoidosis, and further that the longitudinal increase in iNKT cell numbers and the changes in expression of SLAM signaling factors characterize the clinical remission of sarcoidosis. These observations support the hypothesis that the loss of immunoregulatory iNKT cells might contribute to the clinical appearance of sarcoidosis and, vice versa, that recovery of immunoregulatory iNKT cells might contribute to an improvement in symptoms and thus to the clinical remission of sarcoidosis. Because most of the data were based on analysis of patients that had a reversible clinical phenotype and in whom remission occurred within 4 years, further prospective studies on iNKT cells should obviously focus on patients with unrelenting chronic disease that leads to clinically significant organ impairment. Furthermore, further studies are also needed to better understand the mechanistic and possible Th1-related role of iNKT cells in sarcoidosis. Only such data could confirm the biological and functional importance of this specific population of immunoregulatory T cells in immunopathogenesis of sarcoidosis.

## Abbreviations

BALF: Bronchoalveolar lavage fluid
DLCO: Diffusing capacity of the lungs for carbon monoxide
FEV1: Forced expiratory volume in 1 second
FVC: Forced vital capacity
NKT: Natural killer T
sACE: Serum angiotensin-converting enzyme
SAP: SLAM adaptor protein
SLAM: Signaling lymphocytic activation molecule
TCR: T cell receptor
